# Correlation between MRI high-signal parameters and prognosis in cervical spinal cord injury without fracture and dislocation

**DOI:** 10.3389/fneur.2026.1696619

**Published:** 2026-03-27

**Authors:** Sirui Xiao, Xiaokang Cheng, Yuxuan Wu, Chunyang Xu, Hui Yan, Beixi Bao, Jiaguang Tang

**Affiliations:** Department of Orthopaedics, Beijing Tongren Hospital, Capital Medical University, Beijing, China

**Keywords:** axial MRI, axial–sagittal difference ratio (ASDR), cervical spinal cord injury without fracture and dislocation (CSCIwoFD), increased signal intensity (ISI), Japanese Orthopaedic Association (JOA) score, sagittal MRI

## Abstract

**Objective:**

Although patients with cervical spinal cord injury without fracture and dislocation (CSCIwoFD) often exhibit increased signal intensity (ISI) on magnetic resonance imaging (MRI), the prognostic significance of these changes has not been clearly established. This study retrospectively analyzed 118 patients with confirmed CSCIwoFD to examine the relationship between MRI-derived ISI parameters and the recovery rate of Japanese Orthopaedic Association (JOA) scores, in order to evaluate their predictive value for functional outcomes and disease severity.

**Methods:**

A retrospective analysis was conducted on 118 patients diagnosed with cervical spinal cord injury without fracture and dislocation (CSCIwoFD) who underwent surgical treatment at our hospital between August 2020 and June 2024. MRI scans at admission showing high signal intensity changes on axial and sagittal planes were analyzed. Two attending physicians independently measured and recorded the following parameters according to a standardized protocol: Axial values: maximum signal intensity (A_max_), minimum signal intensity (A_min_), mean signal intensity (A_m_) and total spinal cord area (A_t_). Sagittal values: maximum signal intensity (S_max_), minimum signal intensity (S_min_), mean signal intensity (S_m_) and length of high signal intensity (S_1_). Based on previous literature, RR > 50% was defined as good recovery, and RR ≤ 50% as poor recovery. Patients were accordingly classified into good prognosis and poor prognosis groups. The correlation between the above data and postoperative JOA score and improvement rate were analyzed.

**Results:**

All 118 patients successfully underwent surgery. The follow-up period ranged from 12 to 33 months. The mean preoperative JOA score was 8.58 ± 3.02 points. At 3 months postoperatively, the mean JOA score was 13.16 ± 2.22 points, with an average recovery rate of 49.42 ± 23.82%. At 2 years postoperatively, the mean JOA score increased to 15.11 ± 1.53 points, with an average recovery rate of 70.11 ± 28.48%, indicating a definitive therapeutic effect of surgery. When “good recovery at 3 months postoperatively” was used as the grouping variable, the results showed that the ASDR was significantly associated with good recovery (*β* = −0.5101, *p* = 0.036), whereas the other variables were not statistically significant. When “good recovery at 2 years postoperatively” was used as the grouping variable, none of the variables reached statistical significance in the multivariate model. Among them, the ASmax had a regression coefficient of 3.645 (*p* = 0.170), indicating a positive trend that did not reach significance. Using a nonparametric test with “good recovery at 3 months postoperatively” as the grouping variable, the Ad showed a statistically significant difference (*p* = 0.011); ““showed a statistically significant difference (*p* = 0.015); and ““showed a statistically significant difference (*p* = 0.046). Treating the axial–sagittal difference ratio as a single predictive factor, ROC curve analysis yielded an AUC of 0.64. According to the Youden index, the optimal predictive threshold corresponded to a logistic regression probability of 0.157, which approximated an axial–sagittal difference ratio cutoff of 3.06. At this threshold, the model’s sensitivity was 1.00, specificity was 0.32, and the Youden index was 0.32.

**Conclusion:**

This study provides valuable insights into the utility of routine MRI sequences for evaluating CSCIwoFD. The ASDR demonstrated moderate predictive efficacy as a single-variable parameter and may serve as a sensitive screening indicator for early postoperative neurological improvement. Its incorporation into prognostic assessment could offer clinically relevant information and support decision-making. We therefore recommend considering this ratio as a core variable in the construction of early postoperative prognostic models.

## 1 Introduction

Cervical spinal cord injury without fracture and dislocation (CSCIwoFD) represents a special subtype of incomplete cervical spinal cord injury commonly encountered in spinal surgery. Its main characteristics are magnetic resonance imaging (MRI) findings showing spinal cord compression as well as injuries to the intervertebral discs and surrounding soft tissues, while radiographic and computed tomography (CT) examinations reveal no abnormalities of the cervical bony structures or alignment (1). Patients with this condition typically have a clear history of cervical trauma, accompanied by sensory, motor, and autonomic dysfunction. They frequently present with pre-existing conditions such as cervical disc herniation and cervical canal stenosis (2). Following trauma or external force, even in the absence of osseous structural injury, further compression of the spinal cord can occur, resulting in symptoms such as limb weakness, paralysis, numbness, and loss of bladder and bowel control.

Imaging examinations play a crucial role in assessing the condition of patients with spinal cord injury (SCI). For patients with CSCIwoFD, MRI is particularly critical and is considered the gold standard for evaluating the degree of epidural compression, intramedullary tissue injury, and for guiding surgical decision-making after SCI (3, 4). In our hospital’s initial retrospective studies, we found that for CSCIwoFD patients with MRI evidence of spinal cord compression, anterior cervical discectomy and fusion (ACDF) resulted in better neurological recovery than conservative treatment. Subsequent research on surgical timing, combined with partial imaging data assessment, further demonstrated that early surgery provided better outcomes compared with delayed surgery (5). The degree of recovery from cervical spinal cord injury (cSCI) depends largely on the extent of damage to the spinal cord tissue. Regarding increased signal intensity (ISI) observed on MRI, some studies (6) have proposed concepts such as tissue bridges to achieve quantitative measurement. However, quantitative studies specifically addressing the correlation between intramedullary high signal and postoperative neurological recovery remain limited, and to date, no unified standard for measurement has been established. Therefore, in this study, we selected CSCIwoFD patients with MRI evidence of high signal intensity who underwent surgical treatment in our hospital. By measuring and recording the relevant MRI parameters of ISI, and correlating them with Japanese Orthopaedic Association (JOA) scores obtained during follow-up, we aimed to explore the relationship between intramedullary signal changes on MRI and neurological recovery in CSCIwoFD patients.

## 2 Materials and methods

### 2.1 General data

A retrospective analysis was conducted on 118 patients diagnosed with cervical spinal cord injury without fracture and dislocation (CSCIwoFD) who underwent surgical treatment at our hospital between August 2020 and June 2024. Among them, 84 were male and 34 were female, with a mean age of 51.50 ± 12.42 years. Causes of injury included: motor vehicle accidents (38 cases), falls from standing height (38 cases), falls from height (19 cases), and other trauma (25 cases). All patients were followed up for 12–33 months postoperatively.

Inclusion criteria: (1) a definite history of cervical trauma; (2) MRI of the cervical spine showed varying degrees of cervical degeneration and abnormal high signal intensity at the lesion site, while X-ray and CT scans revealed no obvious fracture or dislocation; (3) age between 18 and 80 years; (4) no previous central nervous system or psychiatric disorders; (5) no history of spinal surgery.

Exclusion criteria: (1) concomitant intracranial or thoracic diseases before or after treatment, such as cerebral infarction, cerebral hemorrhage, psychiatric disorders, or cognitive impairment, which could affect spinal cord or neurological function; (2) cervical or other site fractures or traumatic injuries; (3) inability to complete MRI examination; (4) severe osteoporosis, inability to tolerate surgery, or patients opting for conservative treatment; (5) other pathological lesions of the spine; (6) patients lost to follow-up or deceased during outpatient follow-up after surgery.

### 2.2 Imaging data

#### 2.2.1 Examination methods

All patients underwent routine cervical spine radiography, CT, and MRI examinations, which revealed no structural injuries such as cervical fracture or dislocation. MRI examinations were performed using a GE Discovery MR750 3.0 T superconducting MRI system. Sagittal T₂-weighted imaging (T₂WI) parameters: repetition time/echo time/number of excitations (TR/TE/NEX) = 3,000 ms/100 ms/2; slice thickness/slice spacing (ST/SP) = 3.0 mm/1.0 mm. Sagittal T₁-FLAIR parameters: TR/TE/NEX = 2,400 ms/25 ms/2; ST/SP = 3.0 mm/1.0 mm. Axial T₂WI parameters: TR/TE/NEX = 1800 ms/120 ms/4; ST/SP = 1.5 mm/0.5 mm. The scanning range extended from the foramen magnum to the T1 vertebral body (sagittal) and from the C2/3 to C6/7 intervertebral levels (axial).

#### 2.2.2 Measurement methods

**Figure 1:**
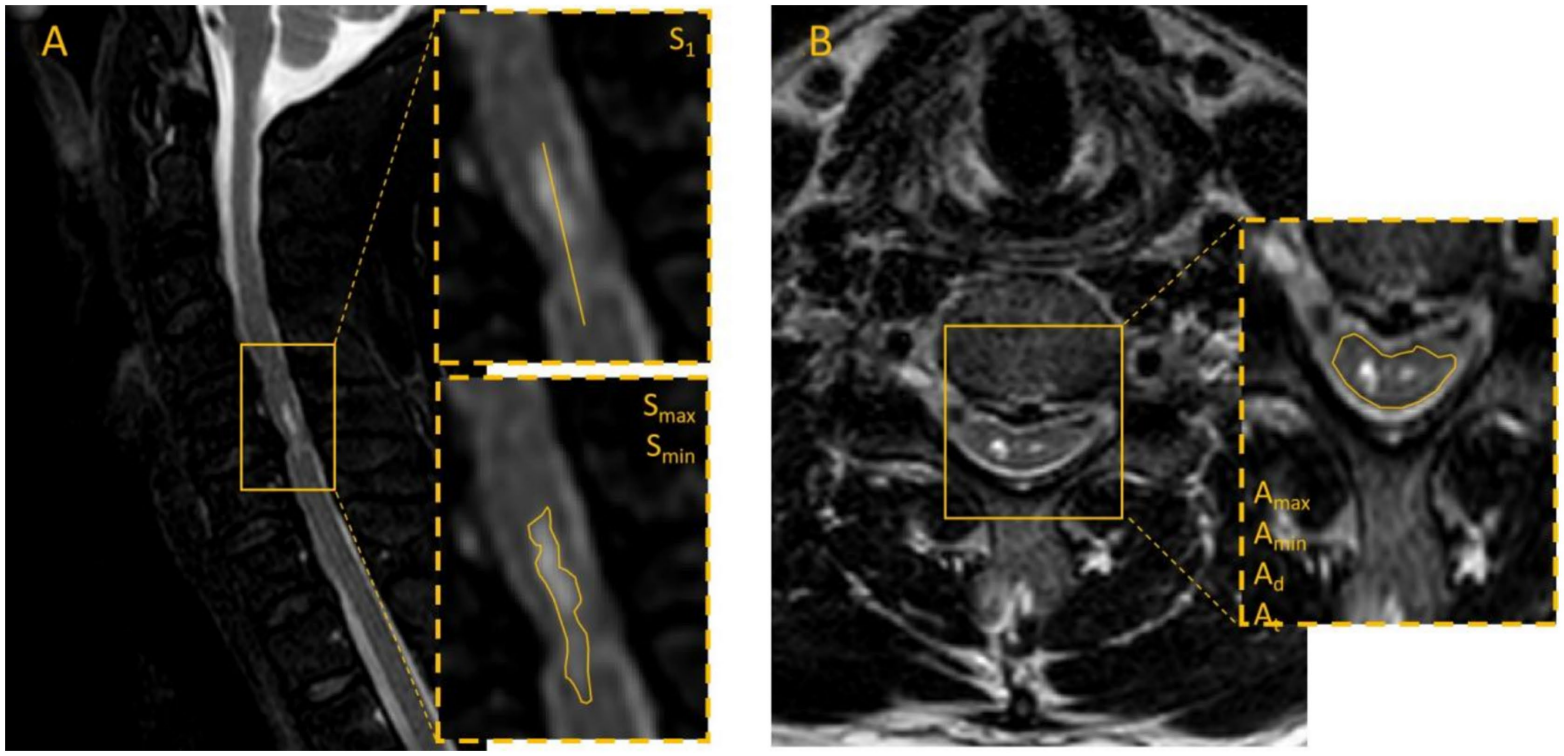
Illustrates the measurement methods for cervical MRI images: **(A)** Sagittal image outlining the high-signal region, measuring its length, and recording relevant values. **(B)** Axial image outlining the spinal cord contour using the image-viewing system, ensuring complete coverage of the cord without including cerebrospinal fluid or other structures, with corresponding signal intensity and area values recorded.

We collected cervical MRI scans from all admitted patients who exhibited intramedullary high-signal changes on both axial and sagittal sequences. Two experienced radiologists in our institution independently performed measurements according to a standardized protocol. The following parameters were recorded: the maximum (A_max_) and minimum (A_min_) intramedullary signal intensities on axial sections, the mean axial signal intensity (A_m_), the total axial spinal cord area (A_t_), the maximum (S_max_) and minimum (S_min_) intramedullary signal intensities on sagittal sections, and the sagittal length of the high-signal lesion (S_1_). Representative measurement procedures are illustrated in Figure 1. The mean values from the two observers were used for analysis. For patients with multisegmental spinal cord injury, the axial slice showing the highest signal intensity was selected for final measurement. The Definitions of MRI signal parameters are shown in Table 1.

**Table 1:** Definitions of MRI signal parameters.

Abbreviation	Definition	Measurement plane/description
Amax	Maximum signal intensity on axial section	Highest intramedullary T2WI value within the lesion
Amin	Minimum signal intensity on axial section	Lowest intramedullary T2WI value within the lesion
Am	Mean axial signal intensity	Average intramedullary signal on axial section
At	Total axial spinal cord area	Outlined spinal cord cross-sectional area (excluding CSF)
Smax	Maximum signal intensity on sagittal section	Highest intramedullary T2WI value within the lesion
Smin	Minimum signal intensity on sagittal section	Lowest intramedullary T2WI value within the lesion
S1	Sagittal length of high-signal lesion	Longitudinal extension of T2 hyperintensity along the spinal cord
Axial difference value	Amax − Amin	Range of signal heterogeneity within the axial plane
Sagittal difference value	Smax − Smin	Range of signal heterogeneity within the sagittal plane
Axial–sagittal difference ratio (ASDR)	(Amax − Amin)/(Smax − Smin)	Relative heterogeneity of signal changes across planes
Axial mean–area ratio (AMR)	Am/At	Mean signal intensity normalized to axial cord area

To ensure the consistency and reproducibility of the measurements, the placement of the Region of Interest (ROI) for MRI signal analysis was standardized. The ROI was carefully delineated by the observers to cover the entire area of the spinal cord without including adjacent structures such as the cerebrospinal fluid (CSF) or vertebrae. The axial and sagittal images were examined to ensure that the ROI was accurately positioned at the level of maximal signal intensity. The placement of the ROI was double-checked for reliability, and any discrepancies between the observers were resolved by consensus. This standardization was critical for minimizing measurement errors and ensuring reproducibility.

Based on these values, we calculated the ratio of maximum signal intensity between axial and sagittal planes (A_max_/S_max_), as the AS_max_ indicates, and the ratio of minimum signal intensity (A_min_/S_min_), as the AS_min_ indicates. The difference between A_max_ and A_min_ was defined as the axial difference value (A_d_), while the difference between Smax and Smin was defined as the sagittal difference value (S_d_). The ratio of the axial difference value to the sagittal difference value was defined as the axial–sagittal difference ratio (ASDR). In addition, the ratio of the mean axial signal intensity (A_m_) to the total axial spinal cord area (A_t_) was calculated and defined as the axial mean-area ratio (AMR).

The following ratios and derived values were calculated:


Axial/Sagittal maximum(ASmax)=(AmaxSmax)×100%

Axial/Sagittal minimum(ASmin)=(AminSmin)×100%

$$\text{Axial difference}\;(A_{d})=A_{\max}-A_{\min}$$

$$\text{Sagittal difference}\;(S_{d})=S_{\max}-S_{\min}$$

$$\begin{array}{l} \text{Axial}-\text{sagittal Difference Ratio}\;(\text{ASDR})\\ =\frac{\left(A_{\max}–A_{\min}\right)}{\left(S_{\max}–S_{\min}\right)}\times 100\%=\frac{A_{d}}{S_{d}}\times 100\% \end{array}$$

Axial Mean−area Ratio(AMR)=AmAt×100%

### 2.3 Treatment protocol

Upon admission, all patients received cervical immobilization with a neck brace, electrocardiographic monitoring, oxygen inhalation, and nutritional support to stabilize vital signs. Preoperative treatments included high-dose steroid pulse therapy, mannitol dehydration therapy, and gastric mucosal protectants.

Surgical approach was determined based on symptoms and MRI findings: Patients with localized anterior spinal cord compression (e.g., disc herniation, ossification of the posterior longitudinal ligament) and 1–2 levels of cervical degeneration underwent anterior cervical discectomy and fusion (ACDF). Patients with multilevel degenerative changes or multilevel ligament ossification underwent posterior cervical procedures, such as laminectomy with internal fixation or expansive laminoplasty. All surgeries were performed or supervised by the same senior spinal surgeon in our hospital. One drainage tube was placed in the cervical wound in each case. Postoperatively, patients continued to use a cervical collar for 2–3 months and received adjuvant therapy, including neurotrophic agents, low-dose steroids, mannitol, gastric mucosal protectants, and rehabilitation training. Outpatient follow-up was conducted at 3 months, 6 months, 1 year, and 2 years postoperatively, including repeat radiography, CT, and MRI.

### 2.4 Outcome measures

Neurological function was assessed at admission and at 3 months, 6 months, 1 year, and 2 years after surgery, using the Japanese Orthopaedic Association (JOA) scoring system for spinal cord function (7), which includes: Upper limb motor function (4 points), Lower limb motor function (4 points), Sensory function of upper/lower limbs and trunk (6 points), Bladder function (3 points). The JOA recovery rate (RR) was calculated according to the method of Yonenobu et al. (8):


$$\mathrm{RR}=[\frac{\left(\mathrm{JOA}\;\text{score}\;\mathrm{at}\;\text{the last follow}\;\mathrm{up}-\mathrm{JOA}\;\text{score}\;\mathrm{at}\;\text{admission}\right)}{\left(17-\mathrm{JOA}\;\text{score}\;\mathrm{at}\;\text{admission}\right)}]\times 100\%$$

Based on previous literature, RR > 50% was defined as good recovery, and RR ≤ 50% as poor recovery. Patients were accordingly classified into good prognosis and poor prognosis groups.

### 2.5 Statistical analysis

All statistical analyses were performed using SPSS version 27.0. Measurement data conforming to a normal distribution were expressed as mean ± standard deviation (x̄ ± s). Univariate and multivariate logistic regression analyses were performed to assess influencing factors. Independent-samples t tests or repeated-measures ANOVA were used for intergroup comparisons of continuous variables. Chi-square (*χ*^2^) tests were used for categorical variables. A *p* value < 0.05 was considered statistically significant.

Baseline characteristics and imaging parameters were compared between the good and poor prognosis groups. Receiver operating characteristic (ROC) curves were plotted, and the area under the curve (AUC) was calculated to evaluate the predictive value of each variable for good prognosis.

## 3 Results

**Figure 2:**
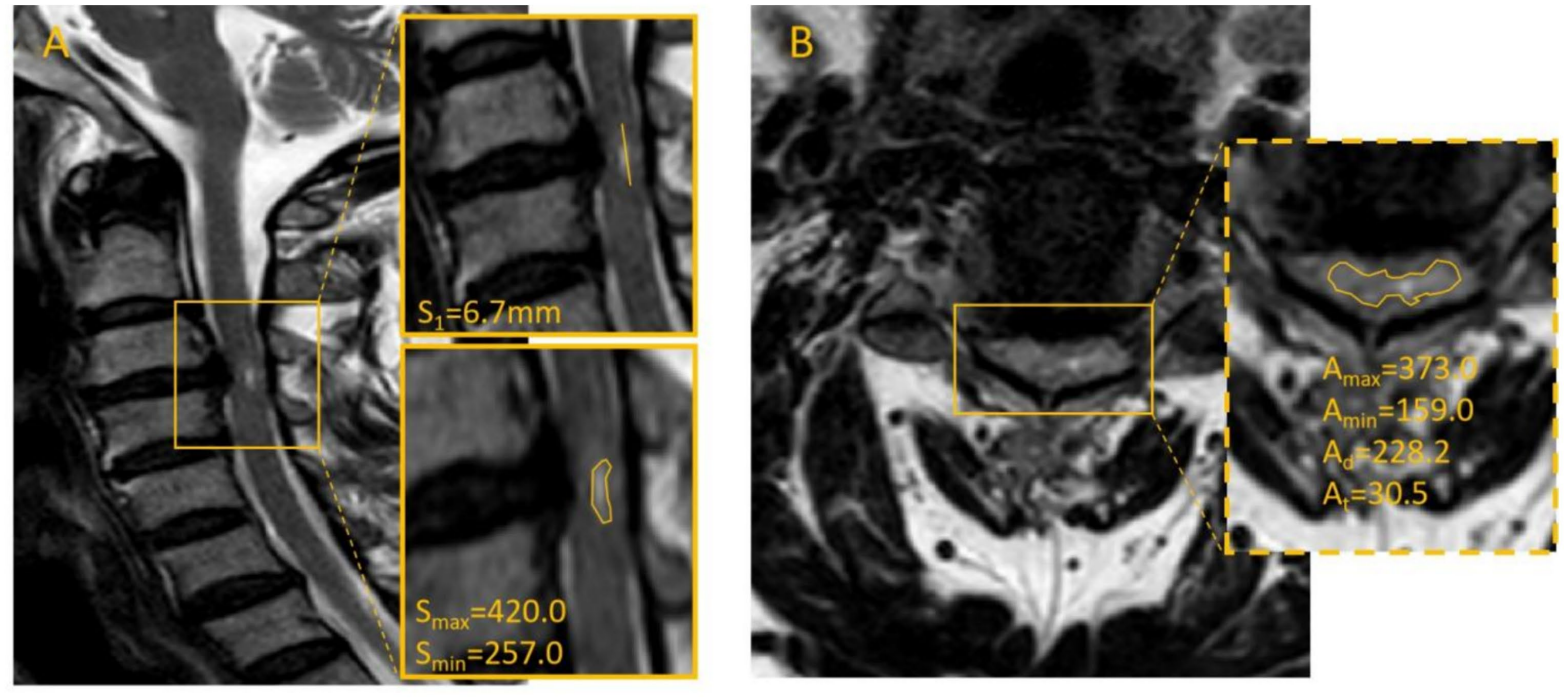
Patient Wu, male, 45 years, admitted 5 days after a motor-vehicle accident. The patient presented with hyperesthesia and decreased muscle strength predominantly in the left limbs; the admission JOA score was 9. The admission cervical MRI is shown in A and B; the measured axial–sagittal difference ratio was 131.29%. The patient underwent single-level ACDF. At 3 months postoperatively, the JOA score was 12 and the RR was 37.5%; at 2 years postoperatively, the JOA score was 16 and the RR was 87.5%.

**Figure 3:**
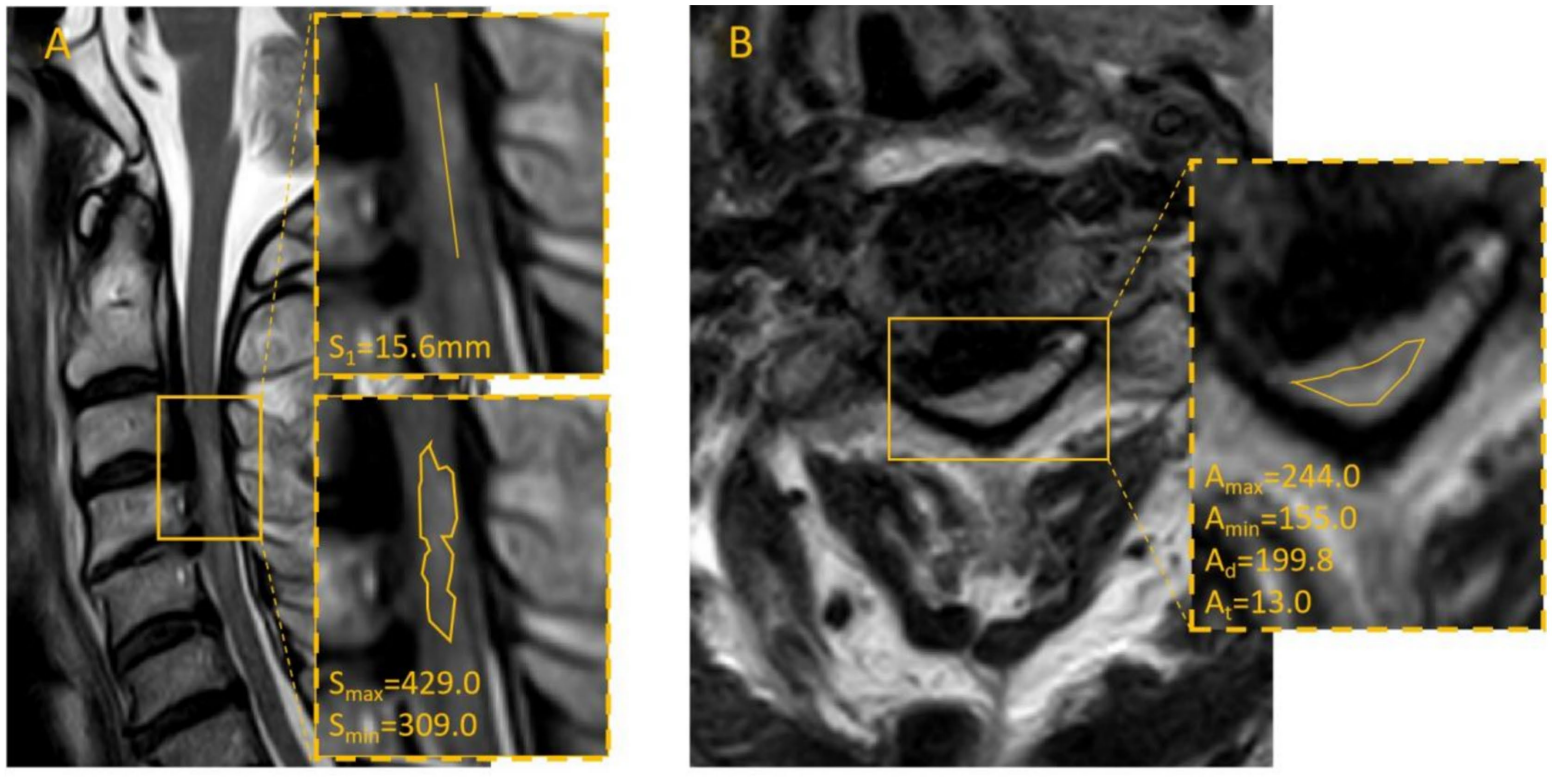
Patient Kang, male, 50years old, admitted 4days after a fall injury. The patient had muscle strength of the four limbs at grade 0–1 with sensoryimpairment; the admission JOA score was 6. The admission cervical MRI is shown in A and B; the measured axial–sagittal difference ratio was 74.17%. The patient underwent five-level posterior cervical surgery. At 3 months postoperatively, the JOA score was 12 and the RR was 54.5%; at 2 years postoperatively, the JOA score was 16 and the RR was 90.9%.

All 118 patients successfully underwent surgery. The follow-up period ranged from 12 to 33 months. The mean preoperative JOA score was 8.58 ± 3.02 points. At 3 months postoperatively, the mean JOA score was 13.16 ± 2.22 points, with an average recovery rate of 49.42 ± 23.82%. At 2 years postoperatively, the mean JOA score increased to 15.11 ± 1.53 points, with an average recovery rate of 70.11 ± 28.48%, indicating a definitive therapeutic effect of surgery. Representative imaging findings of typical cases are shown in Figures 2, 3.

The inter-rater reliability between the two observers was assessed using the intraclass correlation coefficient (ICC), which demonstrated excellent consistency for all measured parameters. The ICC values ranged from 0.85 to 0.93, indicating high reliability across both axial and sagittal MRI measurements. The lower bounds of the 95% confidence intervals for all parameters were above 0.80, further supporting the robustness of the measurements. Additionally, all ICC values were statistically significant, with *p*-values ranging from 0.0001 to 0.0010, demonstrating strong inter-observer agreement. Corresponding results of ICC are shown in Table 2.

**Table 2:** Reliability of measurements between two observers (ICC values).

Variable	ICC	95% CI lower bound	95% CI upper bound	*p*-value
A_max_	0.92	0.89	0.95	0.0001
A_min_	0.89	0.86	0.92	0.0002
A_m_	0.91	0.88	0.93	0.0001
A_t_	0.85	0.82	0.88	0.0010
S_max_	0.93	0.90	0.96	0.0007
S_min_	0.87	0.84	0.90	0.0005
S_m_	0.90	0.87	0.93	0.0003
S_1_	0.83	8.20	0.86	0.0008

### 3.1 Logistic regression analysis

To identify independent prognostic factors influencing postoperative neurological improvement, we constructed a multivariable logistic regression model using imaging signal parameters and analyzed the correlation with JOA score improvement rates at different postoperative time points. When analyzing the improvement rate of the JOA score at 3 months and 2 years postoperatively, we found a partial correlation.

When “good recovery at 3 months” was used as the grouping variable, the results showed that the ASDR was significantly associated with good recovery (*β* = −0.5101, *p* = 0.036). Details are provided in Table 3. This finding suggests that a smaller ratio of axial to sagittal signal difference was associated with better early neurological recovery. Other variables, such as Cmax and S1, showed trends of negative and weak negative correlations with postoperative recovery, respectively, but these did not reach statistical significance. These results indicate that the degree of signal heterogeneity between imaging planes may play an important predictive role in early functional prognosis of patients with CSCIwoFD.

**Table 3:** Logistic regression results for MRI signal parameters predicting 3-month JOA recovery.

Variable	Coefficient (β)	Standard Error	*Z*-value	*p*-value	95% CI Lower	95% CI Upper
A_max_	−0.012	0.008	−1.46	0.143	−0.027	0.004
A_min_	0.001	0.012	0.01	0.995	−0.023	0.023
A_m_	0.015	0.014	1.08	0.278	−0.012	0.042
A_t_	−0.041	0.031	−1.34	0.180	−0.102	0.019
S_max_	−0.023	0.021	−1.1	0.270	−0.065	0.018
S_min_	−0.010	0.017	−0.6	0.551	−0.042	0.023
S_m_	0.036	0.035	1.03	0.304	−0.032	0.104
S_1_	0.012	0.045	0.26	0.795	−0.076	0.099
AS_max_	1.643	1.370	1.2	0.230	−1.0421	4.3287
Ad	−0.003	0.003	−0.83	0.301	−0.0088	0.0036
AMR	0.015	0.035	0.71	0.056	−0.0437	0.093
ASDR	−0.510	0.342	−1.49	0.036	−1.1806	0.1604

When “good recovery at 2 years” was used as the grouping variable, none of the variables reached statistical significance in the multivariate model. Among them, the “axial/sagittal maximum” had a regression coefficient of 3.645 (*p* = 0.170), showing a positive trend and suggesting a possible association with long-term recovery, though not statistically significant. The “ASDR” (*β* = −0.0859, *p* = 0.411) and other signal parameters also did not show independent predictive value. These findings suggest that some parameters with predictive value at 3 months may have diminished predictive efficacy at 2 years. The corresponding results are shown in Table 4.

**Table 4:** Logistic regression results for MRI signal parameters predicting 2-years JOA recovery.

Variable	Coefficient (β)	Standard Error	*Z*-value	*p*-value	95% CI Lower	95% CI Upper
A_max_	−0.0097	0.018	−1.01	0.277	−0.071	0.007
A_min_	−0.0091	0.008	0.23	0.552	−0.035	0.043
A_m_	0.0173	0.018	0.99	0.3245	−0.017	0.052
A_t_	−0.0433	0.047	−0.92	0.3577	−0.136	0.049
S_max_	−0.0321	0.031	−1.04	0.2993	−0.093	0.0285
S_min_	0.009	0.052	0.17	0.8631	−0.093	0.1109
S_m_	0.0352	0.050	0.7	0.4827	−0.063	0.1337
S_1_	0.0617	0.095	0.65	0.5147	−0.124	0.2472
AS_max_	4.188	2.629	1.59	0.1111	−0.96	9.34
Ad	−0.006	0.01	−0.63	0.41	−0.014	0.0316
AMR	−0.0497	0.034	−1.48	0.1388	−0.116	0.0161
ASDR	−0.4823	0.315	−1.53	0.0859	−1.1	0.1353

### 3.2 Intergroup comparisons

A nonparametric test was conducted using “good recovery at 3 months” as the grouping variable. Good prognosis group: 44 males, 18 females; mean age 51.72 ± 13.99 years. Causes of injury: motor vehicle accidents (19 cases), falls from standing height (20 cases), falls from height (9 cases), and other trauma (14 cases). Poor prognosis group: 40 males, 16 females; mean age 51.29 ± 10.94 years. Causes of injury: motor vehicle accidents (17 cases), falls from standing height (18 cases), falls from height (10 cases), and other trauma (11 cases). When analyzed with “good recovery at 3 months” as the grouping variable, nonparametric testing indicated that the axial difference was statistically significant (*p* = 0.011). Additional parameters also demonstrated statistical significance (*p* = 0.015, *p* = 0.046, respectively), with specific results shown in Table 5.

**Table 5:** Nonparemetric comparison by 3-month neurological recovery status.

Variable		Good prognosis group (Group A, *n* = 62)	Poor prognosis group (Group B, *n* = 56)	P score
Gender	Male	44	40	0.462
	Female	18	16	
Age		51.72 ± 13.99	51.29 ± 10.94	0.888
Reason (n)	Traffic	19	17	0.525
	Fall injury	20	18	
	Fall injury from high	9	10	
	Others	14	11	
A_max_		435.56 ± 152.89	405.82 ± 154.23	0.386
A_min_		182.32 ± 130.24	190.35 ± 111.21	0.233
A_m_		288.48 ± 114.73	292.58 ± 113.22	0.171
A_t_		41.28 ± 12.71	33.71 ± 16.13	0.224
S_max_		411.80 ± 186.26	389.12 ± 165.72	0.693
S_min_		278.60 ± 128.69	263.12 ± 148.25	0.574
S_1_		12.48 ± 2.08	10.24 ± 4.10	0.832
AS_min_		62.92 ± 25.16	78.12 ± 33.74	0.089
AS_max_		105.52 ± 31.23	101.76 ± 23.57	0.522
A_d_		253.24 ± 98.41	215.47 ± 113.02	0.011
S_d_		133.20 ± 71.94	126.00 ± 66.53	0.062
Axial mean-area ratio (AMR)		6.99 ± 1.27	8.68 ± 1.77	0.015
Axial–sagittal difference ratio (ASDR)		87.64 ± 41.79	117.12 ± 54.26	0.046

**Figure 4:**
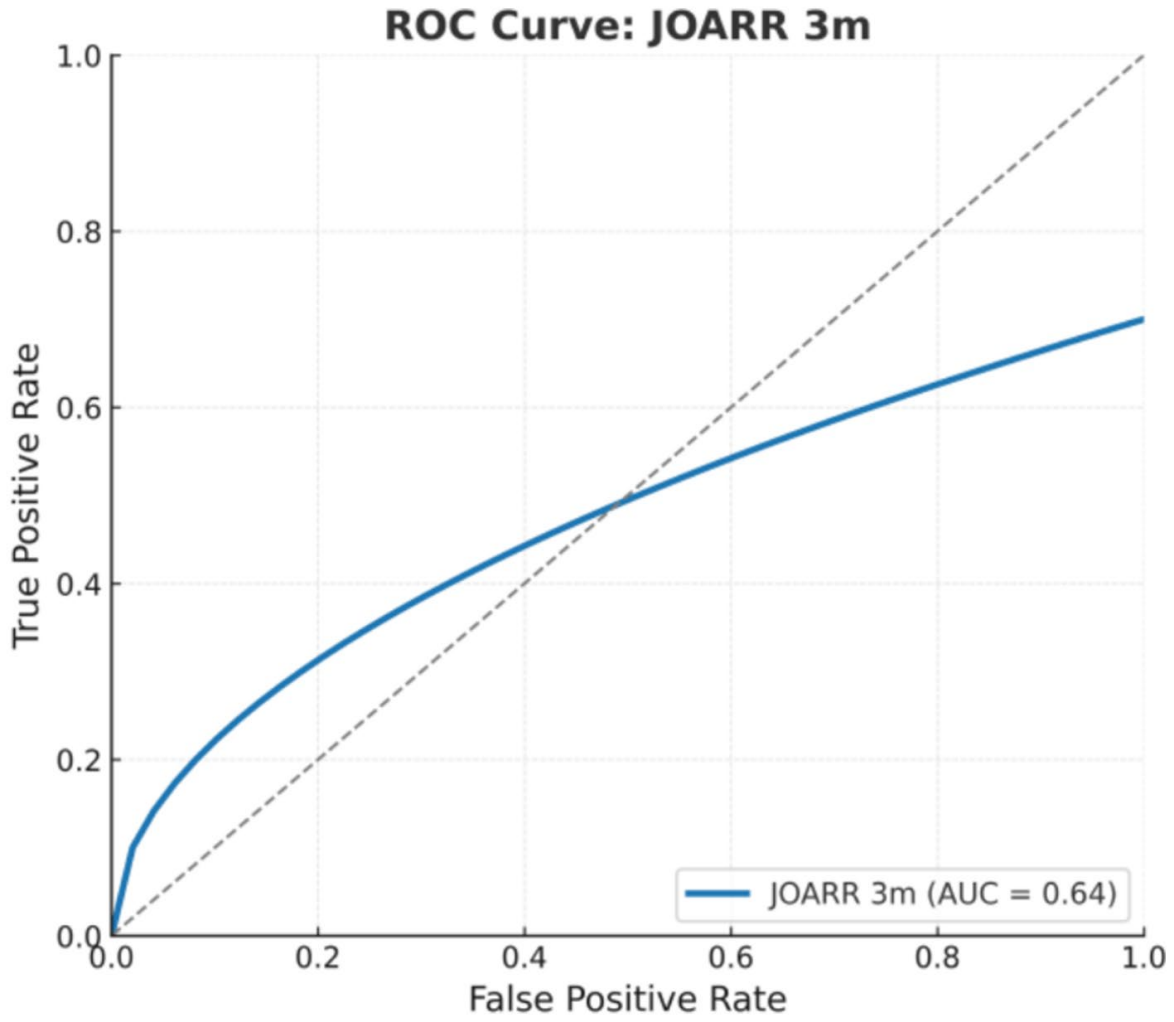
ROC curve: JOA recovery rate at 3 months (JOARR 3 m). Receiver operating characteristic (ROC) curve for the logistic regression model based on the transverse-sagittal signal difference ratio, assessing the ability to predict favorable neurological recovery at 3 months postoperatively. The area under the curve (AUC) was 0.64 (95% CI, 0.51–0.76), indicating moderate predictive performance.

Based on logistic regression results, the ASDR was further analyzed as a single predictive factor using ROC curve analysis to assess its predictive ability for 3-month neurological recovery. The results demonstrated an AUC of 0.64, indicating moderate discriminatory power. The optimal cutoff threshold determined by the Youden index was a logistic regression probability of 0.157, corresponding to an ASDR of approximately 3.06. At this threshold, the model achieved a sensitivity of 1.00, specificity of 0.32, and a Youden index of 0.32. These results indicate that the ASDR, as a single predictor, had certain predictive efficacy and statistical significance. The results are illustrated in Figure 4.

**Figure 5:**
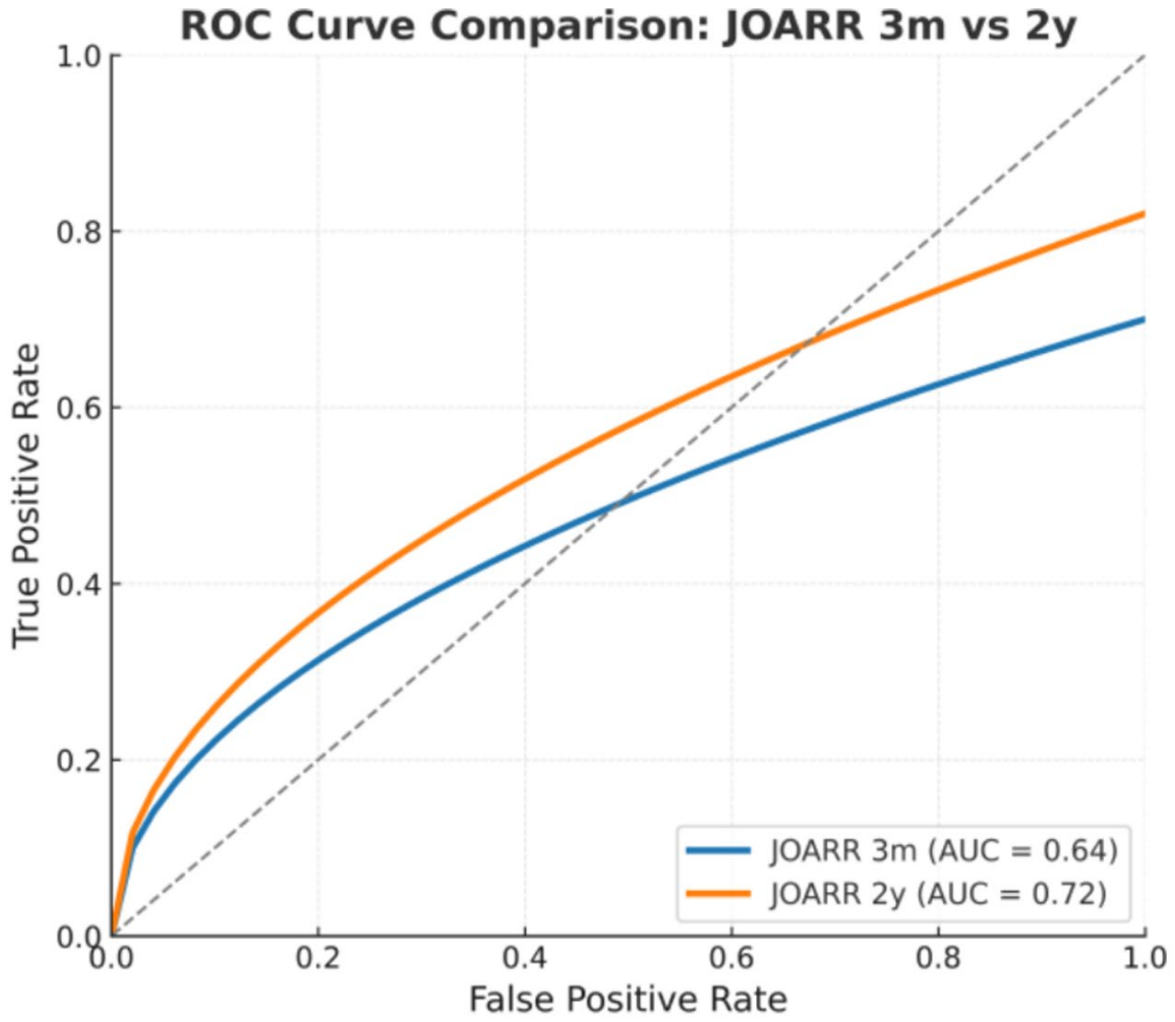
Comparative ROC curves: JOA recovery at 3 months vs. 2 years (JOARR 3 m vs. 2y) comparison of ROC curves for predicting favorable neurological recovery at 3 months and 2 years after surgery. The model for 3-month recovery achieved an AUC of 0.64 (95% CI: 0.51–0.76), whereas the 2-year model showed superior performance with an AUC of 0.72 (95% CI: 0.60–0.83). The difference between the two models reached borderline statistical significance (DeLong test, *p* = 0.048), suggesting a higher predictive value of the transverse-sagittal signal difference ratio for long-term outcomes.

To compare the predictive performance of the transverse-sagittal signal difference ratio for favorable neurological recovery at both 3 months and 2 years postoperatively, we additionally generated an ROC curve based on the 2-year JOA recovery rate. The results demonstrated that the model predicting favorable recovery at 3 months showed moderate discriminative ability, with an area under the curve (AUC) of 0.64 (95% CI: 0.51–0.76). In contrast, the model predicting favorable recovery at 2 years performed better, achieving an AUC of 0.72 (95% CI: 0.60–0.83). A borderline statistically significant difference was observed between the two models (DeLong test, *p* = 0.048). These findings suggest that the transverse-sagittal signal difference ratio has predictive value for early functional recovery and demonstrates superior predictive performance for long-term neurological outcomes, indicating its potential clinical utility. The results are illustrated in Figures 4, 5.

## 4 Discussion

Neurological recovery following traumatic spinal cord injury (SCI) critically influences acute treatment decisions, prognostic counseling, functional goal setting, and individualized rehabilitation planning. Thus, the ability to predict neurological outcomes after SCI is of paramount importance. The prognostic value of increased signal intensity (ISI) on MRI has been well established (9). However, in the existing literature, the quantitative relationship between MRI high signal values and prognosis in patients with cervical spinal cord injury without fracture and dislocation (CSCIwoFD) remains insufficiently explored. Most previous studies have focused primarily on the mere presence of T2 hyperintensity or its morphological classification. For instance, a CSFM scoring system incorporating “increased spinal cord signal intensity” has been proposed (10), which reflects the degree of compression by the posterior longitudinal ligament and helps predict prognosis in patients with ossification of the posterior longitudinal ligament (OPLL). Similarly, the CCCFLS system (11), based on the grade and number of spinal cord signal abnormalities, has been suggested to correlate with the severity of clinical symptoms. However, with regard to SCI—and more specifically CSCIwoFD—quantitative studies assessing the direct relationship between ISI and functional outcome scores remain limited (6, 12).

In the present study, we systematically analyzed MRI-based signal intensity alterations in both axial and sagittal planes in 118 CSCIwoFD patients, along with cross-sectional area and sagittal lesion length at compressed segments. Special emphasis was placed on their differences and ratios, which were then correlated with the postoperative recovery rate (RR) of the JOA score. By defining RR > 50% as good recovery and RR ≤ 50% as poor recovery, we added “favorable neurological improvement” as an evaluative endpoint. Our findings revealed that both univariate and multivariate logistic regression analyses identified the ASDR as an independent predictor of early (3-month) neurological recovery (*β* = −0.510, *p* ≈ 0.036). ROC analysis yielded an AUC of 0.64, with a sensitivity of 100% and specificity of 32% at the cutoff value of approximately 3.06. These results suggest that a smaller axial-sagittal signal difference ratio is associated with better early postoperative neurological recovery, highlighting its potential as a prognostic marker for short-term outcomes in CSCIwoFD patients. In contrast, parameters such as Cmax and sagittal lesion length demonstrated limited prognostic utility. Notably, the predictive value of all parameters diminished during the 2-year follow-up, underscoring the need for further refinement of long-term prognostic indicators.

A possible explanation is that the first 3 months post-surgery typically represent the acute and subacute recovery phase. Prior studies (13, 14) have indicated that neurological recovery during this period mainly depends on spontaneous reversible changes following the initial injury, such as resolution of edema, relief of conduction block, restoration of neuronal excitability, and limited plastic repair. The ASDR reflects the relative discrepancy between axial and sagittal hyperintensity, serving as a quantification of spatial distribution and heterogeneity of lesions. A smaller ratio suggests more localized or longitudinally extended but less transverse disruption, implying relative preservation of transverse spinal cord structures (particularly long tracts) and therefore greater potential for early recovery. By contrast, in the chronic phase (1–2 years postoperatively), neurological improvement relies more on long-term rehabilitation, neural regeneration, synaptic plasticity, and compensatory mechanisms (15). These processes outweigh the structural damage observable on initial imaging. Consequently, patients with slower early recovery may still achieve compensatory functional improvement through rehabilitation, whereas those with favorable early recovery may plateau due to chronic inflammation, glial scarring, or limited axonal regeneration (16). Thus, the ASDR primarily reflects the reversible structural substrate in the acute phase but is insufficient to predict long-term recovery trajectories. Moreover, long-term recovery outcomes are influenced by non-imaging factors such as age, comorbidities, rehabilitation adherence, and lifestyle (17, 18). These variables were not incorporated into our model, which may further dilute the predictive role of the ASDR in long-term prognosis.

Axial and sagittal signal changes represent different dimensions of injury. Axial differences reflect local transverse intramedullary hemorrhage or edema, while sagittal differences indicate longitudinal lesion extension. The ASDR may therefore represent lesion heterogeneity—that is, the relative relationship between transverse and longitudinal signal dispersion. A lower ratio reflects minimal transverse variability but marked longitudinal spread, suggesting primarily vertical dissemination with preserved transverse integrity. This pattern implies better preservation of neural tracts and thus greater early recovery potential. Conversely, a higher ratio indicates marked transverse injury, often associated with intramedullary hemorrhage or diffuse edema, leading to greater tract disruption and worse prognosis.

T2-weighted imaging (T2WI) was selected as the basis for MRI signal quantification due to its high sensitivity in detecting pathological changes after SCI and its strong clinical utility (19). T2WI is particularly sensitive to water content alterations, clearly delineating spinal cord edema, intramedullary hemorrhage, ischemic softening, and demyelination—all secondary pathological processes closely associated with prognosis. In contrast, T1-weighted imaging (T1WI) is less sensitive to acute edema and minor hemorrhage, limiting its value for lesion boundary identification or quantitative analysis in the acute phase. T1WI is more suitable for chronic conditions such as syringomyelia, fatty degeneration, or old hemorrhage, rather than acute prognostic evaluation. Therefore, the use of T2WI in our study ensured optimal contrast and reproducibility for capturing signal alterations relevant to functional recovery, consistent with prevailing research and clinical practice.

T2 hyperintensity typically manifests as increased signal within axial cross-sections and longitudinal streaks on sagittal images. Prior studies (20) suggest that hyperintensity exceeding 3 cm or accompanied by >1 cm intramedullary hemorrhage predicts poor prognosis. In cervical spondylosis, preoperative MRI hyperintensity correlates with a 15% reduction in JOA scores and decreased quality of life postoperatively, potentially reflecting ischemia and demyelination from chronic compression (21). However, hyperintensity alone does not fully represent spinal cord integrity. Some studies (6) have described “tissue bridges,” visible as relatively hypointense narrow bands of intact axonal fibers traversing the lesion on sagittal T2WI. The width of these bridges may serve as an imaging biomarker of preserved neural continuity. While hyperintensity reflects irreversible injury and inflammatory edema, tissue bridges indicate residual tract continuity and recovery potential. Our ASDR essentially quantifies signal dispersion across planes, where smaller values may reflect concentrated hyperintensity with preserved surrounding tracts—conceptually aligned with the tissue bridge hypothesis. Thus, incorporating both the shape and distribution of hyperintensity provides a more comprehensive structural-functional understanding of SCI, strengthening prognostic accuracy.

Despite the common use of sagittal T2 hyperintensity length as a prognostic measure, reliance on lesion length alone has limitations (22). Lesion length cannot distinguish between edema versus necrosis, nor reliably predict tract preservation. Some patients with long hyperintensities retain neural integrity, while others with short hyperintensities sustain severe axonal disruption. Furthermore, lesion length is influenced by nonspecific processes (edema, ischemia, demyelination, gliosis) and technical parameters (slice thickness, angle, image quality), reducing reliability. In contrast, the ASDR better reflects the three-dimensional distribution and heterogeneity of injury. This ratio aligns with concepts such as tissue bridge width and cross-sectional compression area, suggesting that future imaging-based prognostic models should integrate multi-parametric and structural-functional dimensions rather than relying solely on lesion length.

Although CSCIwoFD lacks overt fractures or dislocations, patients frequently exhibit severe neurological deficits. This suggests that the pathophysiological mechanism lies in intrinsic biomechanical and microstructural injury to the spinal cord. CSCIwoFD typically occurs in individuals with pre-existing degenerative canal stenosis (2). In these patients, spinal cord reserve space is limited, making it vulnerable to transient “pincer-like” compression or shear stress during sudden flexion, extension, rotation, or axial loading. These forces, though insufficient to fracture bony structures, can disrupt the blood–spinal cord barrier, cause microvascular rupture, edema, ischemia–reperfusion injury, and apoptosis.

At the neuronal level, early injury induces axonal degeneration, demyelination, and glial activation, leading to focal conduction block. The central gray matter, particularly vulnerable to hypoxia, undergoes necrosis, appearing as hyperintensity on T2WI (23). Axonal transection promotes Wallerian degeneration and inflammatory propagation, exacerbating secondary injury cascades (oxidative stress, excitotoxicity, inflammatory infiltration). Meanwhile, partially spared fibers under mild traction or compression may remain detectable as hypointense “bridges” on sagittal T2WI, suggesting residual structural continuity and recovery potential.

Thus, the neuropathology of CSCIwoFD is highly heterogeneous, comprising varying degrees of axonal loss, demyelination, and inflammation. Hyperintensity largely reflects irreversible injury, whereas hypointense bridges signify preserved pathways that may underlie recovery. The ASDR, by quantifying the three-dimensional relationship of hyper- and hypointense regions, encapsulates this heterogeneity, supporting the development of more precise imaging-based prognostic models and guiding postoperative rehabilitation.

Previous studies have generally interpreted MRI hyperintensity as indicative of poor prognosis, with particular focus on its presence and length. For instance, maximum lesion cross-sectional area and lesion length have been identified as significant prognostic risk factors in pediatric SCIWORA (20), with ROC AUCs of 0.91 and 0.78, respectively, both negatively correlating with recovery. Other studies (21) argue that conventional MRI cannot distinguish reversible edema from irreversible contusion, limiting reliability. In our study, isolated parameters such as maximum/minimum axial or sagittal signal values, lesion area, and lesion length showed limited prognostic value—consistent with prior literature. By contrast, the ASDR effectively predicted early recovery, likely because it integrates inter-planar heterogeneity and reflects three-dimensional lesion distribution, offering superior sensitivity. In this study, we developed a predictive model based on the transverse-sagittal signal difference ratio to evaluate postoperative neurological recovery and assessed its performance at two time points: 3 months and 2 years after surgery. Receiver operating characteristic (ROC) analysis demonstrated moderate predictive performance for favorable recovery at 3 months (AUC = 0.64, 95% CI: 0.51–0.76), whereas the model showed better discrimination for predicting favorable recovery at 2 years (AUC = 0.72, 95% CI: 0.60–0.83). A borderline statistically significant difference was observed between the two models (DeLong test, *p* = 0.048), indicating that the transverse-sagittal signal difference ratio may hold clinical value as an early prognostic marker. However, although the transverse-sagittal signal difference ratio demonstrated significant predictive value for early recovery, signal-related variables failed to serve as independent predictors for long-term outcomes. Further analyses revealed no statistically significant differences in long-term neurological recovery between groups, suggesting that long-term outcomes are likely influenced by multiple contributing factors beyond MRI-based signal parameters. Future studies should therefore include larger sample sizes and adopt more refined functional assessments, such as the ASIA motor score and ASIA sensory score, to separately evaluate the relationships between long-term recovery, motor function, and sensory function, thereby providing deeper insights into the mechanisms underlying neurological restoration.

Neurological recovery following cervical spinal cord injury without fracture and dislocation (CSCIwoFD) is influenced by multiple factors, including the baseline severity of the injury, comorbid conditions, and clinical management. While the Area of Spinal Cord Signal-to-Disk Ratio (ASDR) has demonstrated a high sensitivity, its relatively low specificity suggests its limitations as an independent predictor. ASDR, in its current form, may not fully account for the complex interplay of clinical and imaging factors that contribute to recovery. This may also explain why no positive results were obtained in the long-term outcomes, such as the JOA score improvement rate at 2 years postoperatively, in this study. This highlights the need for a more comprehensive approach that integrates multiple variables in predicting long-term outcomes. Future studies will incorporate additional MRI modalities and parameters, such as diffusion tensor imaging (DTI), to develop more robust and precise prognostic models for neurological recovery in CSCIwoFD patients. This approach will better account for the multifaceted nature of recovery, improving both the sensitivity and specificity of predictive indicators.

In summary, the ASDR demonstrated prognostic efficacy for early postoperative outcomes, reflecting the distribution of reversible injury and preserved neural structures. Nevertheless, its independent predictive value in long-term recovery remains limited due to the complexity of biological mechanisms and individual variability. Future studies should integrate this ratio with structural, functional, and clinical parameters to establish multi-factorial, stage-specific prognostic models capable of dynamically predicting recovery trajectories.

This study has several limitations. First, it is a retrospective single-center study with a relatively small sample size and without stratification by age or injury severity. Second, MRI measurements were performed manually; although repeated by multiple observers to minimize bias, inter-observer variability remains. AI-assisted analysis could enhance accuracy in future work. Third, imaging data were limited to static MRI, excluding spinal dynamics and advanced modalities such as diffusion tensor imaging (DTI). Future studies should incorporate DWI, DTI, dynamic MRI, and delayed MRI to construct more comprehensive predictive models.

## 5 Conclusion

This study provides valuable insights into the utility of routine MRI sequences for evaluating CSCIwoFD. The ASDR demonstrated moderate predictive efficacy as a single-variable parameter and may serve as a sensitive screening indicator for early postoperative neurological improvement. Its incorporation into prognostic assessment could offer clinically relevant information and support decision-making. We therefore recommend considering this ratio as a core variable in the construction of early postoperative prognostic models.

## Data Availability

The original contributions presented in the study are included in the article/supplementary material, further inquiries can be directed to the corresponding author.
